# Evaluation and Source Analysis of Plant Heavy Metal Pollution in Kalamaili Mountain Nature Reserve

**DOI:** 10.3390/plants14101521

**Published:** 2025-05-19

**Authors:** Jialin Li, Abdugheni Abliz, Buasi Nueraihemaiti, Dongping Guo, Xianhe Liu

**Affiliations:** 1 College of Geography and Remote Sensing Sciences, Xinjiang University, Urumqi 830046, China; lijialin@stu.xju.edu.cn (J.L.); asiyaa@stu.xju.edu.cn (B.N.); dongpingguo@stu.xju.edu.cn (D.G.); liuxianhe@stu.xju.edu.cn (X.L.); 2 Xinjiang Key Laboratory of Oasis Ecology, Xinjiang University, Urumqi 830046, China; 3 Xinjiang Field Scientific Observation and Research Station for the Oasisization Process in the Hinterland of the Taklamakan Desert, Yutian 848400, China

**Keywords:** plant heavy metals, pollution assessment, source analysis, APCS-MLR modeling, random forest modeling

## Abstract

Plants serve as vital components of ecosystems, with their contamination status acting as sensitive indicators of environmental pollution. Therefore, the precise assessment of plant heavy metal contamination and source identification are crucial for regional ecological conservation and sustainable development. This study investigated heavy metal pollution in four characteristic plant species (*Anabasis aphylla* L., *Alhagi camelorum* Fisch., *Reaumuria songonica* (PalL)Maxim., and *Haloxylon ammodendron* (C. A. Mey.) Bunge.) within the Kalamaili Mountain Nature Reserve, employing comprehensive methodologies including pollution indices, bioconcentration factors (BCFs), absolute principal component score–multiple linear regression (APCS-MLR), and the random forest model (RF). The key findings revealed the following: The soil exhibited severe Cd and Hg contamination. The plant Cr concentrations exceeded standard limits by 31.89 to 147 fold. The Pb, Hg, and As content in plants showed significant differences. The plants displayed differential metal enrichment capacities, ranked as Cr (BCF = 3.28) > Hg (1.22) > Cd (0.92) > Cu (0.25) > Zn (0.15) > Pb (0.125) > As (0.125), highlighting Cr, Hg, and Cd as priority ecological hazards. Complex interactions were observed, with *Reaumuria songonica* (PalL)Maxim. showing strong Cd soil–plant correlation (r = 0.78), whereas *Alhagi camelorum* Fisch. demonstrated negative associations (Cd: r = −0.21). APCS-MLR identified mining/smelting as primary contributors to Cd (63.49%), Zn (55.66%), and Cr (45.51%), while transportation dominated Pb emissions (72.92%). Mercury pollution originated from mixed sources (56.18%), likely involving atmospheric deposition, and RF modeling corroborated these patterns, confirming industrial and transportation synergies for Cd, Zn, Cr, Cu, Hg, and As, with Pb predominantly linked to vehicular emissions. This multidisciplinary approach provides scientific evidence for establishing heavy metal monitoring systems and formulating targeted remediation strategies in arid ecologically fragile regions.

## 1. Introduction

As the main component of the ecosystem, plants provide nutrients for the biosphere, and their growth and development directly affect the entire ecosystem [1]. With the advancement of industrialization, the scale of production activities, such as mineral extraction, is rapidly expanding, resulting in large amounts of heavy metals entering the soil and vegetation of the surrounding areas along with atmospheric deposition, precipitation, and surface water [2,3], leading to increasing heavy metal pollution in the surrounding areas [4]. Heavy metals enter into the roots, stems, leaves, and seeds of plants as they grow and develop, hindering plant productivity and transferring enrichment through the food chain, posing a threat to animal and human health [5]. Soil and sediments act as sinks for heavy metal pollutants [6], while water, soil, and air act as transport media for heavy metals [7], influencing the transportation and accumulation of heavy metals. Currently, more studies have been carried out on soil- and water-related heavy metal pollution, evaluation, and source resolution [8], while plants act as receptors of heavy metal pollution [9], and most of the current research on plant heavy metals focuses on how to alleviate the stress of heavy metal pollution on plants [10,11,12], with relatively few studies focusing on the evaluation of plant heavy metal pollution and source resolution. In addition, as the soil–plant system is an important heavy metal transport and accumulation system, there are fewer studies on the migration and transport behaviors of heavy metals within the system [13]; therefore, it is necessary to carry out scientific pollution evaluations and source analyses of plant heavy metals and to carry out research on the accumulation and transport of heavy metals in the soil–plant system.

Indicators used for heavy metal pollution evaluation in current relevant studies include the geo-accumulation index (Igeo) [14], potential ecological risk index (RI) [15], pollution load index (PLI) [16], Nemerow pollution index (NPI) [17], etc. The above indicators are commonly used to evaluate the degree of pollution of soil and water and to measure the ecological hazards of heavy metals in plants, which can be combined with their enrichment capacity for a comprehensive analysis. In addition, accurately grasping the source of heavy metal pollution in the region is crucial for regional environmental management, and many scholars at home and abroad have carried out work related to the analysis of heavy metal sources in plants, with the results showing that mining activities [18], sewage discharge [19], transportation activities [20] and other human activities, as well as natural factors such as the geological background [21], can lead to heavy metal pollution in plants. Heavy metal pollution source allocation methods can be divided into two categories: source identification and source quantification [22]. The identification of pollution sources mainly relies on methods such as GIS and principal component analysis [23,24,25], while source quantification uses receptor models for quantitative analysis [26,27]. Sun et al. [28]. explored the sources of heavy metals in a wetland using a PMF model, showing that pesticides, fertilizers, livestock manure, sewage irrigation, agricultural sources, and soil matrix can all lead to heavy metal pollution and affect plants. Skorbiłowicz et al. [29] used a cluster analysis and other human methods to analyze the heavy metal contamination of the Narew watershed and aquatic plants, which mainly originated from sewage treatment plants, transportation activities, and radioactive waste repositories. Zacharie et al. [30] used a principal component analysis and a hierarchical cluster analysis to investigate the heavy metal contamination of the Lekie River watershed, which mainly originated from the mining of ores and the discharge of industrial wastewater. Adnan et al. [31] analyzed the heavy metals in the area around a smelter based on the PMF and PCA methods and concluded that the heavy metals in the area around the smelter mainly originated from industrial activities, atmospheric deposition, and soil matrices. Most of the previous studies used receptor modeling for source quantification, but heavy metal pollution often presents a complex superimposed effect of natural geologic background and anthropogenic activity inputs, and it is difficult to accurately quantify the pollution contribution using traditional single assessment methods [32]. The random forest model, as a robust nonparametric method, can effectively process complex data, discover hidden nonlinear relationships, and develop predictive models [33] and has been widely used in recent years in the fields of identification, prediction, and monitoring of pollutants in environmental media [34,35]. In the current study, the random forest model and the APCS-MLR receptor model will be used for source resolution, and as machine learning is not easily affected by the data-processing process [36], the efficient and convenient features of the APCS-MLR model [37] will be utilized to explore the sources of plant heavy metal pollution.

As the only hoofed wildlife activity area in the northwest of China, the ecological environment of the Kalamaili Mountain Nature Reserve is closely linked to various types of wildlife in the reserve. However, due to the combined constraints of climate, geographic location, and other factors, the reserve is characterized by sparse vegetation and the fragile ecological environment [38]. As a typical arid desert area, the Kalamaili Mountain Nature Reserve is an ecologically fragile area, has a low environmental carrying capacity, and is difficult to repair after damage, thus making it a serious ecological protection situation. In addition, the southern foot of the reserve is an industrial-intensive area with various types of industrial industries, and industrial activities, such as mining activities, can cause heavy metal pollution in the area 100 km away from the mining area [39]. In this context, the environment of the protected area is affected by the neighboring industrial activities, which affects the ecological balance of soil and plants and limits the green development of the protected area’s ecology. In this study, we will take the Kalamaili Mountain Nature Reserve as the study area, select representative plants in the reserve as the study objects, and utilize the NPI and RI to evaluate the risk of heavy metal pollution status of soils and plants in the study area, as well as utilize enrichment coefficients and correlation coefficients to parse out the linkages between soil–vegetation systems. In addition, the APCS-MLR model and RF model were used to analyze the sources of heavy metal pollution in plants. The results of this study will clarify the ecological risk of vegetation under the disturbance of industrial and mining activities and provide theoretical support for optimizing the ecological maintenance and control strategy of the protected area.

## 2. Results

### 2.1. Descriptive Statistics of Soil–Plant Heavy Metal Contents

As can be seen from Table 1, the average contents of heavy metals Cr, Cu, Zn, Cd, Pb, Hg, and As in the soil were 44.38, 20.89, 55.95, 0.13, 17.5, 0.0093, and 6.64 mg·kg^−1^, respectively, and in terms of the average contents of heavy metals, the content of Cd exceeded the background value of Xinjiang soil by 1.08 times. The mean values of Cr, Cu, Zn, Cd, Pb, Hg, and As did not exceed the screening values when compared with the risk screening values of the national standard (GB/15618-2018). The coefficients of variation of each heavy metal in the soil were 35.10%, 47.85%, 22.53%, 45.34%, 11.03%, 41.64%, and 59.13%, which were all of medium variation. From the coefficients of variation, it can be seen that As and Cu have higher degrees of variation and are more affected by external influences. Table A1 shows the significant comparative differences between the soil contents of each heavy metal and the background values in Xinjiang. Among them, the *p*-values of Zn, Pb, Hg, and As were all 1, while the *p*-values of Cr, Cu, and Cd were 0.9183, 0.9934, and 0.1994, respectively, which indicated that there was no significant difference between the seven heavy metal elements in the study area and the background values of Xinjiang, whereas the element Cd had a relatively significant difference from the background values.

As can be seen from Table 2, the average content of seven heavy metals Cr, Cu, Zn, Cd, Pb, Hg, and As in *Anabasis aphylla* L. were categorized as 294.24, 6.51, 11.63, 0.17, 1.91, 0.01, and 1.53 mg·kg^−1^, of which Cr was 147 times the standard limit value, and Hg, Cu, Zn, Cd, Pb, and As did not exceed the standard limit value; The average content of seven heavy metals—Cr, Cu, Zn, Cd, Pb, Hg, and As—in *Alhagi camelorum* Fisch. was 70.10, 3.78, 4.89, 0.06, 0.65, 0.01, and 0.46 mg·kg^−1^, of which Cr was 35 times the standard limit value; the average content of seven heavy metals—Cr, Cu, Zn, Cd, Pb, Hg, and As—in *Reaumuria songonica* (PalL)Maxim. was 115.46, 5.86, 8.31, 0.12, 3.65, 0.01, and 0.92 mg·kg^−1^, of which Cr and Pb were 57.73 and 1.22 times the standard limit values, respectively; and the average contents of seven heavy metals—Cu, Zn, Cd, Pb, Hg, and As—in *Haloxylon ammodendron* (C. A. Mey.) Bunge. were 63.79, 3.85, 7.48, 0.08, 0.39, 0.01, and 0.26, of which Cr was 31.89 times the standard limit. From the above, it can be seen that Cr exceeds the standard in the above four plants, which has a greater ecological risk. The coefficients of variation of the heavy metal contents in each plant were large, indicating that the factors affecting the heavy metal contents in plants are complicated.

As shown in Table A2, the Cr, Cu, Zn, Pb, and As contents were significantly higher than the standard limit (*p* = 0.00001), except for Hg (*p* = 0.3699). Table A3 shows that the concentrations of Cr, Cu, Zn, and Cd did not differ significantly among the four plants (*p* > 0.05), while the concentrations of Pb, Hg, and As differed significantly (*p* < 0.05). Further two-by-two comparisons (in Table A4) showed that, with regard to the element Pb: *Anabasis aphylla* L.—*Haloxylon ammodendron* (C. A. Mey.) Bunge. differed significantly (*p* = 0.0079), as did *Reaumuria songonica* (PalL)Maxim.—*Haloxylon ammodendron* (C. A. Mey.) Bunge. (*p* = 0.0063). For the element Hg, *Reaumuria songonica* (PalL)Maxim.—*Haloxylon ammodendron* (C. A. Mey.) Bunge. differed significantly (*p* = 0.0098). With regard to the element As, *Anabasis aphylla* L.—*Haloxylon ammodendron* (C. A. Mey.) Bunge. (*p* = 0.0145) and *Reaumuria songonica* (PalL)Maxim.—*Haloxylon ammodendron* (C. A. Mey.) Bunge. (*p* = 0.0096) were the main sources of difference. In summary, the plant heavy metal accumulation characteristics were regulated by species specificity. The interspecific differences in Pb and As were mainly driven by the comparison of *Anabasis aphylla* L., *Reaumuria songonica* (PalL)Maxim. and *Haloxylon ammodendron* (C. A. Mey.) Bunge., while the differences in Hg were concentrated in *Reaumuria songonica* (PalL)Maxim. and *Haloxylon ammodendron* (C. A. Mey.) Bunge.

**Figure 1:**
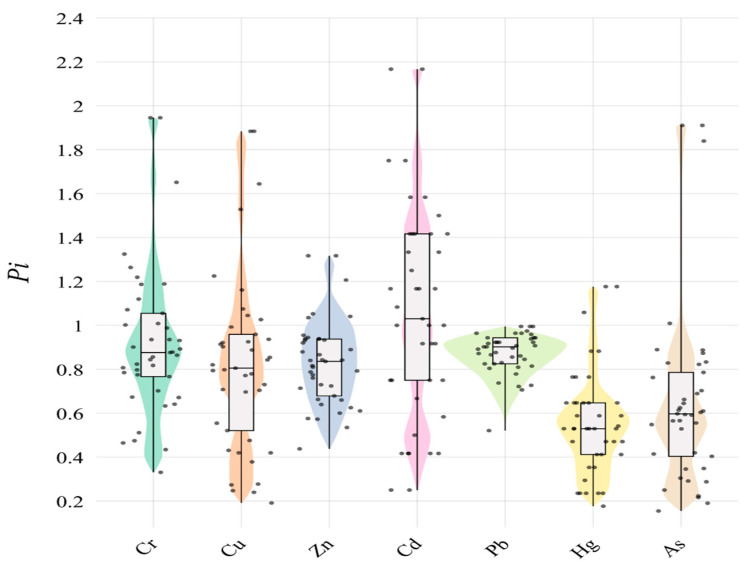
The P*i* of soil heavy metal.

The Nemerow pollution index (Table 3 and Figure 1) shows that Cr, Cu, Zn, Cd, and As are in a moderate state of pollution, while Pb and Hg are in a warning limit.

From the composite potential ecological risk index (Table 4), it can be seen that Cr and Zn in the soil are at the slight ecological hazard level; Cu, Pb, and As are at the medium ecological hazard level; and Cd and Hg are at the very strong ecological hazard level. Combining the NIPI with the RI shows that Cr, Cu, Cd, Hg, and Pb are more seriously polluted.

### 2.2. Plant–Soil Heavy Metal Correlation Analysis

**Figure 2:**
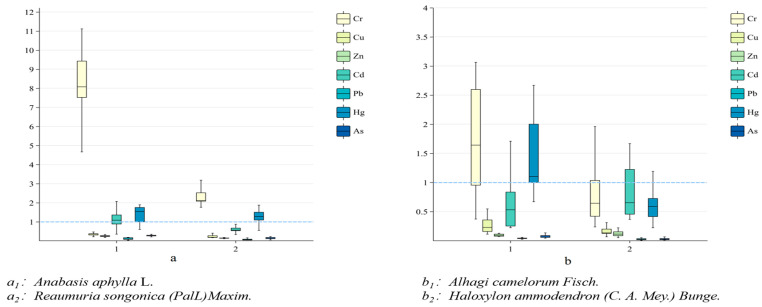
BCF for different plants. Note: a, b no practical significance, only to show more clearly the different enrichment capacities of the four plants.

In order to express the interrelationship between plant heavy metals and soil heavy metals more accurately, the interconnection between plants and soil at the same sampling site will be analyzed. Analyzing the interconnections between soil and plants can help provide a basis for subsequent heavy metal pollution management. The BCF can intuitively respond to the uptake and migration capacity of plants for soil heavy metals, and the uptake capacity of the same plant for different heavy metal elements and the uptake capacity of different plants for the same element are also somewhat different [50]. As can be seen from Figure 2, the BCF for Cr were greater than 1 for *Anabasis aphylla* L. (7.303), *Alhagi camelorum* Fisch. (1.78), *Reaumuria songonica* (PalL)Maxim. (2.60), and *Haloxylon ammodendron* (C. A. Mey.) Bunge. (1.45), which indicated that Cr could be easily absorbed by these four types of plants ((${\bar{BCF\;}}_{Cr}$) = 3.28). Regarding the BCF of Hg, the BCF of *Anabasis aphylla* L. (1.35), *Alhagi camelorum* Fisch. (1.46), and *Reaumuria songonica* (PalL)Maxim. (1.26) were all greater than 1, and only *Haloxylon ammodendron* (C. A. Mey.) Bunge. (0.82 < 1) indicated that Hg was also easily absorbed by the plants and was enriched in the body of the plants ((${\bar{BCF\;}}_{Hg}$) =1.22). The BCF of Cd in *Anabasis aphylla* L. (1.424 > 1), *Alhagi camelorum* Fisch. (0.71), *Reaumuria songonica* (PalL)Maxim. (0.71), and *Haloxylon ammodendron* (C. A. Mey.) Bunge. (0.84) indicated that the effect of Cd on plants should not be neglected ((${\bar{BCF\;}}_{Cr}$) =0.92), not being easily enriched in plants and having less effects on plants. The differences in the heavy metal enrichment ability of different plants in the same soil environment indicate that the external environment was not a decisive factor for heavy metal accumulation. Studies have shown that the coexistence of multiple heavy metals in the soil environment of the same region is different from the effect of a single heavy metal in the soil on plants, and the enrichment phenomenon of plants can be antagonistic or synergistic due to the presence of multiple heavy metals [51,52,53].

**Figure 3:**
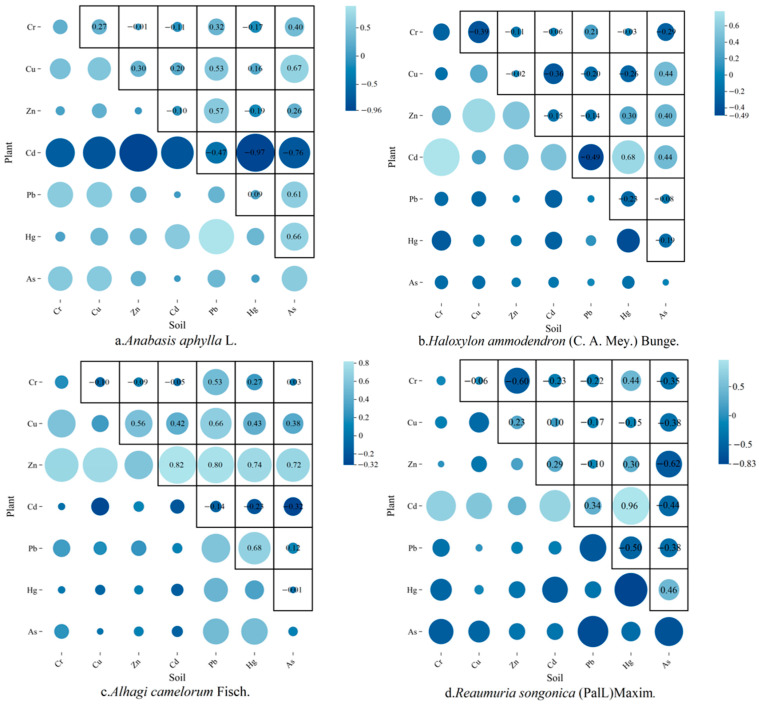
Correlation coefficients between different plants and soil.

The correlation coefficients can effectively reflect the correlation between soil heavy metals and plant heavy metals, visualize the transfer efficiency of pollutants between environmental media, and explain the main sources of heavy metals in plants. In Figure 3, the correlations between seven heavy metals in four plant species and in soil are demonstrated. In *Anabasis aphylla* L., Cd-plant showed a very strong negative correlation (r = −0.967) with Hg-soil, suggesting that soil Hg contamination may inhibit the phytosorption of Cd or that the two are competitive; Cu-plant showed a significant positive correlation (r = 0.67) with As-soil, reflecting the fact that As can modulate and promote the translocation of Cu, whereas the correlation between Pb-plant and Pb-soil was only weakly correlated (r = 0.313), indicating that its accumulation was dominated by other factors. For *Alhagi camelorum* Fisch., Zn-plant showed strong positive correlations with Cd-soil (r = 0.82), Pb-soil (r = 0.795), and Hg-soil (r = 0.739), implying that Zn promotes multi-metal co-transport [54,55], and Cd-plant showed no significant correlation with Cd-soil (r = −0.208), indicating that its accumulation is dominated by other factors and thus indicating that its uptake is regulated by non-soil factors (e.g., atmospheric deposition). In *Reaumuria songonica* (PalL)Maxim., Cd-plant showed a strong positive correlation (r = 0.96) with Hg-soil, indicating that soil Hg contamination could promote Cd uptake in *Reaumuria songonica* (PalL)Maxim., which was diametrically opposed to *Anabasis aphylla* L., and demonstrated that the uptake of the same heavy metal varied among different plants, whereas As-plant showed a strong negative correlation (r = −0.761) with Pb-soil, which may be due to Pb As-plant being strongly negatively correlated with Pb-soil (r = −0.761), probably due to the inhibition of As enrichment by Pb; Cr-plant was negatively correlated with Zn-soil (r = −0.602), indicating that excess Zn would lead to the blockage of Cr uptake. In *Haloxylon ammodendron* (C. A. Mey.) Bunge., Cd-plant was positively correlated with Cr-soil (r = 0.778), indicating that Cr contamination in the soil may promote Cd accumulation in the plant, while Pb-plant was weakly correlated with soil Hg-soil (r = −0.226), suggesting that the two uptake pathways do not interfere with each other.

Heavy metal accumulation in plants was driven not only by soil concentrations but also by synergistic or antagonistic interactions between elements, such as the “bridging effect” of Zn in *Alhagi camelorum* Fisch. and the strong antagonism of Cd-Hg in *Anabasis aphylla* L. The low correlation of some elements (e.g., Cd in *Alhagi camelorum* Fisch.) with soil concentrations suggests that stomatal uptake, microbial activity, or genetic regulation may dominate the accumulation process [56].

The correlation coefficients and BCFs between soil heavy metals and plant heavy metals are key indicators for revealing the migration and transformation of pollutants in the soil–plant system, which is of great significance for ecological risk assessment and pollution control. Combining the BCFs and correlation coefficient, it can be found that the Cd in *Haloxylon ammodendron* (C. A. Mey.) Bunge. and *Reaumuria songonica* (PalL)Maxim. is positively correlated with the Cd content in the soil, with the BCF close to 1, suggesting that Cd is easily enriched and that Cd can have harmful effects on plants; in addition, the Zn in *Haloxylon ammodendron* (C. A. Mey.) Bunge., the Cu and As in *Anabasis aphylla* L., as well as the Zn and Pb in the *Anabasis aphylla* L. are positively correlated with the corresponding elements in the soil, but the enrichment of the above four types of elements in those plants are smaller, indicating that although the appeal of the three species of plants is sensitive to the content of Zn, Cu, As, and Pb in the soil, the accumulation ability is weak. These four species of plants are easily enriched by Cr elements, but the Cr in plants and the Cr in soil show a non-significant correlation, indicating that the four species of plants show extremely strong enrichment of Cr, which is not related to the soil content, which can thus be used for subsequent remediation of contamination [57].

### 2.3. Source Identification by APCS-MLR

The APCS-MLR model was used to analyze the sources of heavy metals in the above plants. Using a principal component analysis, the two main factor scores were converted to absolute principal factor scores, and multiple linear regression analyses were carried out, with the content of heavy metal elements in the measured vegetation as the dependent variable and the absolute factor scores as the independent variables, and the results are shown in Table A5. R^2^ is used to measure the correlation between the model and the actual observations, and the closer the value of R^2^ is to 1, the better the linear fit and the better the simulation results [58]. In this study, the R^2^ of the regression model for each element was greater than 0.5, and only the R^2^ of Pb was slightly lower, at 0.53. The results showed that the model had a better simulation effect.

**Figure 4:**
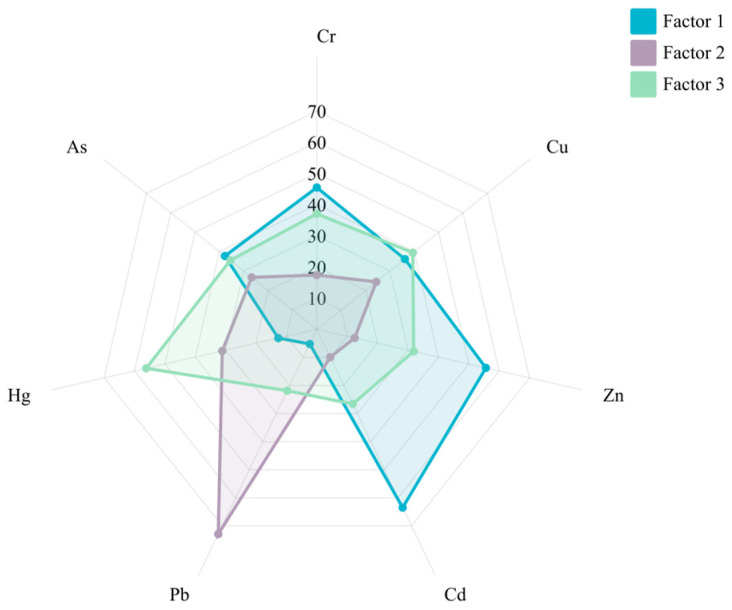
Contribution of plant heavy metal pollution source factors by the APCS-MLR model. Note: Factor 1 refers to the first potential impact factor; factor 2 refers to the second potential impact factor; and factor 3 refers to the third potential impact factor.

The APCS-MLR model identified the main factors that had a greater effect on plant heavy metals based on a principal component analysis. The principal component analysis generated a four-dimensional model based on eigenvalues greater than 1, explaining 71.6% of the variance in the dataset. Among them, APCS1 explained 55.24% of the variance and consisted of Cr, Cu, Zn, Cd, and As, with a loading factor ≥ 0.64 (Table 5), which is a strong loading [59]. All of the above elements were positively correlated, and the significant correlation between heavy metal concentrations indicates that these elements have a common source [60]. From Figure 4, it can be seen that factor 1 has a large contribution to the elements Cd, Zn, and Cr with 63.49%, 55.66%, and 45.51%, respectively, as well as a large effect on Cu and As. Some studies have shown that As enters the environment with metal mining and smelting activities [61,62], and in China, As is mainly associated with Cu, Cu, and Sn deposits [63], and mining activities and smelting likewise cause rapid changes in the element Cd [64,65]. Therefore, factor 1 was initially determined to be mineral mining and smelting activities.

APCS2 explained 16.35% of the variance, including Cu, Pb, Hg, and As, of which the Hg and Pb loading coefficients reached 0.77 versus 0.74, and combined with Figure 4, it was concluded that factor 2 was closely related to the source of the element Pb, with a contribution of 72.92%. This study shows that gasoline combustion leads to an increase in Pb content [66], which determines that factor 2 is a transportation source.

When the APCS-MLR model was utilized to resolve the pollution sources, a class of unknown sources was resolved. Combined with Figure 4, it was concluded that unknown source 3 had a relatively high degree of association with elemental Hg, with a contribution rate of 56.18%. The enrichment of elemental Hg is usually considered to be dominated by mining pollution sources [67], and the Hg in the roots of woody trees and shrubs is mainly absorbed from the soil, while that in the aboveground part is mainly absorbed from the atmosphere [68,69], from which it can be deduced that unknown source 3 is a mixed source of complex origins.

### 2.4. Random Forest Modeling to Predict Pollution Sources

**Figure A1:**
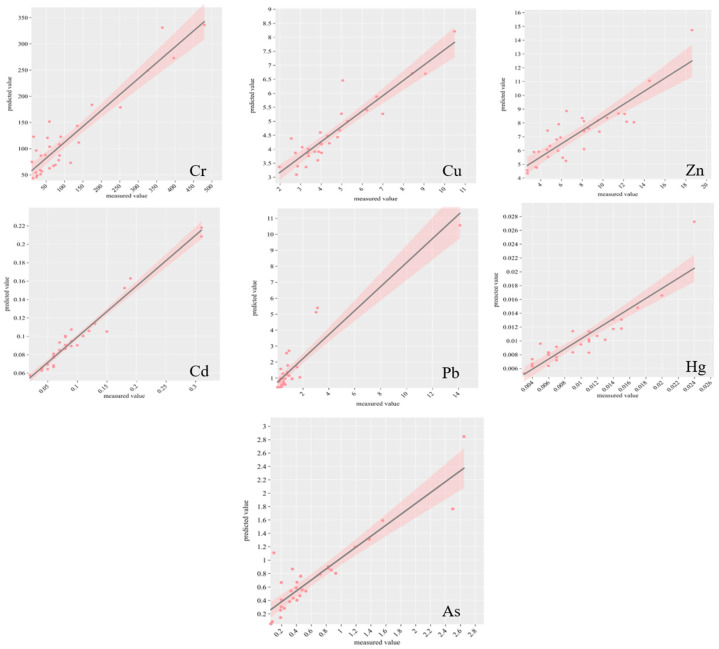
Scatter plot of the RF model’s measured and predicted values.

The RF model prediction performance will be evaluated by R^2^, RMSE, MAE, and RPD, with R^2^ > 0.5 meaning that the model simulation performance is good [70]; the model fit is shown in Figure A1. According to Table 6, which assesses the predictive effectiveness of the RF model, the model showed good statistical significance for most of the heavy metal elements. In terms of goodness of fit (R^2^), the R^2^ values of the training set range from 0.72 (Zn) to 0.85 (Pb), indicating the model’s ability to explain data variability. In the test set, the R^2^ values of Cd (0.86), Cu (0.82), and As (0.81) are higher than or close to those of the training set. This demonstrates the model’s excellent generalization ability. However, the R^2^ value for Zn in the test set drops significantly to 0.54. This suggests that the model’s predictive ability may be limited by feature selection or differences in the data distribution. Regarding error analysis, the RMSE and MAE of the test set are generally lower than those of the training set (e.g., the RMSE of Cu decreases from 0.94 to 0.45, and the MAE of Pb decreases from 0.46 to 0.16). This indicates that the model has high prediction accuracy and stability for most elements. However, the RMSE of the Cr test set (58.88) is slightly lower than that of the training set, and the absolute error remains high. This may reflect data noise or a large concentration span. Regarding model consistency (RPD), the high RPD values of Cd (2.66), Cu (2.35), and As (2.27) suggest robust and reliable predictions, while the low RPD values of Zn (1.48) and Cr (1.78) imply unstable predictions. Taken together, the model’s predictions for elements such as Cd, Cu, Pb, and As are statistically significant (R^2^ ≥ 0.75 for the test set) and suitable for practical scenarios, such as pollution a source analysis or risk assessment. However, Zn predictions need further optimization; introducing relevant environmental factors or adjusting the model structure may be necessary. Overall, the model is valuable in environmental science but needs improvement.

**Figure 5:**
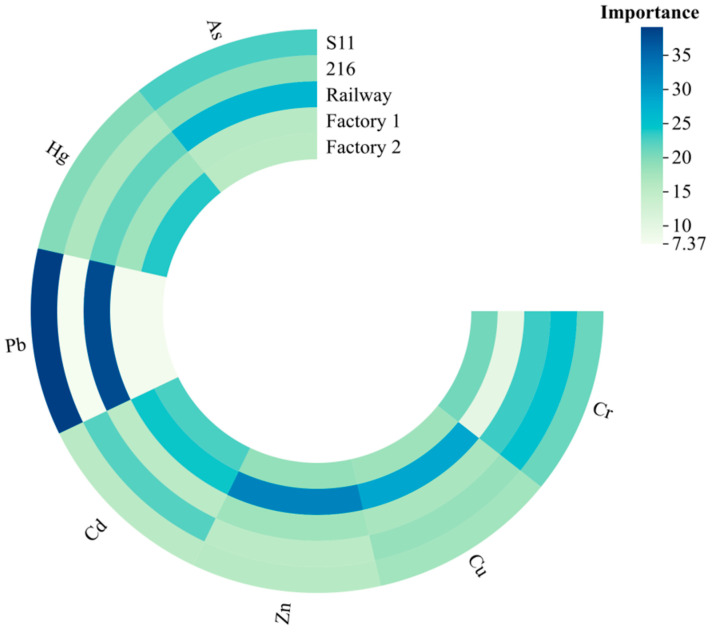
Proportion of importance of different pollution sources in the RF model.

The results of the RF model pollution source analysis are shown in Figure 5. As a whole, the two roads, railroad and S11, contributed the largest percentage. For Cr elemental pollution sources, 216 provincial road contributes, the most with 25.32%; factory 1 contributes the least, with 9.55%; and the remaining three sources account for 20.78 to 23.14. For Cu, factory 1 contributes the most, with 28.70%, and the other four sources account for 17.13 to 18.53. For Zn, factory 1 contributes the most, with 32.28%, and the other four sources account for the range of 17.13 to 18.53. For Zn, the largest contribution is from factory 1, with 32.28%, and the other 4 sources range from 15.36 to 18.76. The sources of Cd are more complex, with 24.24%, 22.68%, and 22.16% from factory 1, factory 2, and Provincial Highway 216, respectively, and 15.40% and 15.52% from railroads and S11, respectively, for the rest. For Pb, the greater contribution is made by railroads and S11, with 37.99% and 39.15%, both of which account for the majority of the pollution sources. The greater contribution of elemental Hg is made by plant 2 and railroads, with 23.91% and 21.54%, respectively, while the remaining three sources range from 16.81 to 19.82. For As, the greatest contribution is made by Railroad and S11, with 26.82% and 22.77%, and the remaining 3 sources range from 15.79 to 18.97.

Overall, the synergistic increase in the contributions of Cr, Zn, and Cd in the factory 1 and factory 2 areas indicates that all three elements are influenced by industrial activities, whereas the close association of the elements Pb and As with S11 suggests that the transportation activities have a greater impact on them. The remaining elements, Hg and Cu, are influenced by a combination of industrial production and transportation.

## 3. Discussion

### 3.1. Characterization of Soil–Plant Heavy Metal Pollution and Ecological Risks

The most serious levels of Cd and Hg contamination were found in the soils of the Kalamaili Mountains Nature Reserve, with potential ecological risk indices of 1205 and 844.71, respectively, which belonged to the serious risk classes. This conclusion is consistent with the ecological risk assessment system proposed by Hakanson, in which highly toxic elements can cause significant ecological hazards even at low concentrations [71]. Cr in plants poses a high ecological risk, exceeding the standard limits by 31.89 to 147 times. Khalili et al. [72] investigating the feasibility of cereals and quinoa as bioindicators of heavy metal pollution in soil using factorial pot experiments, concluded that wheat was highly susceptible to the accumulation of Pb, Cr, and Zn; that all four plant species in the study area were susceptible to the accumulation of Cr and Hg; and Cd easily accumulated in *Anabasis aphylla* L., indicating that different plants have different accumulation capacities for different heavy metals. Chen et al. [73] analyzed the heavy metal content in vegetables in Baotou, Inner Mongolia, and showed that the uptake and translocation of heavy metals were affected by environmental factors, resulting in different concentrations in different vegetable species. The results of Anwar et al. [74] showed that plants produce a chelating agent (a class of enzymatically synthesized cysteine-rich polypeptides that form complexes with a variety of metals), which limits the entry of metal ions into root cells through chelate sequestration. Furthermore, Panda et al. [75] showed that the accumulation of heavy metals in plants is influenced by the exchangeable state of the soil, as well as by a variety of transporter proteins and adaptive mechanisms in the plant. The results of Millaleo and Proshad et al. [76,77] also showed that the uptake of heavy metals by plants depends on the elemental content of the sediment and the physiological environment of the plant. The results of the present study also showed that the uptake and translocation of the same heavy metals varied among plants. In addition, the present study found a strong correlation between plant heavy metal content and soil heavy metal content, which is consistent with the findings of Lu et al. [78]. The low correlation between heavy metal contents between some plants and soil indicated that their uptake was dominated by non-soil factors [79]. The heavy metal contents of the four plants in the study area are subject to variation, attributable to the combined influence of intrinsic plant factors and external factors. A targeted approach to treatment, informed by the unique characteristics of each plant, can be employed to mitigate subsequent pollution. For instance, the planting of *Reaumuria songonica* (PalL)Maxim. and *Haloxylon ammodendron* (C. A. Mey.) Bunge in the area with severe Cd pollution can be reduced to mitigate harm to organisms. Additionally, the utilization of heavy metal elements with high correlation to the soil–plant system (e.g., Zn, As, etc.) can serve as indicator markers of the entire soil–plant system, thereby revealing the current status of the ecological environment.

### 3.2. Analysis of Soil–Plant Heavy Metal Pollution Sources

The joint application of APCS-MLR and RF provided complementary perspectives for the quantification of pollution sources. The APCS-MLR analysis showed that the contribution of industrial activity sources to Cd, Zn, and Cr was more consistent with the regional industrial layout, while the dominant role of transportation sources for Pb was consistent with the findings of Huang et al. on the emission of Pb from transportation activities [80]. The RF model further verified the composite effect of industrial activities and transportation sources, but its predictive performance for Zn was low, probably due to the dispersed importance of the model features due to the diversity of Zn sources. Yuan et al. [81] suggested in their study that animal manure also causes Zn pollution, and combined with the biological characteristics of the study area, it can be concluded that Zn is most likely to be affected by a combination of factors such as industry and animal manure. Shaikh et al. [82] pointed out in their study that the heavy metal contamination of plants may originate from sediments, industrial activities, and other human activities. Du et al. [83] pointed out that Cr contamination can be influenced by both electroplating and diagenesis. In this study, combining the conclusions of the APCS-MLR model with the analysis of the contribution of RF to the potential sources of contamination, we concluded that Cr contamination is mainly influenced by industrial activities. For the source of Hg contamination, Wu and Talukder et al. [84,85] all showed that industrial activities, sewage irrigation, domestic waste, and coal combustion for heating can lead to Hg contamination, and this study advocated that Hg comes from a mixed source of contamination based on the special geographical location of the study area, where both industrial activities and coal combustion have a strong influence. Regarding the source of Cd, Fu et al. [86] showed that industrial pollution, soil erosion, atmospheric deposition, and agricultural activities all contribute to Cd pollution. Since agricultural activities cannot be carried out in the study area and the surrounding area is characterized by intensive industrial activities, the possibility of Cd contamination due to industrial activities is higher. Tong et al. [87] showed that industrial activities such as mining and smelting can lead to the cumulative contamination of Cu and As in the area, and in the light of the actual situation of the study area, the Cu and As contamination of the study area also originated from industrial activities such as mining and smelting. In summary, the soil–plant in the study area is mainly affected by industrial activities and their subsidiary activities. Heavy metal pollutants, generated by industrial activities, migrate into the soil and plants in the study area via transportation, atmosphere, and other pathways. Additionally, plants’ own transit enrichment of heavy metals further leads to the emergence of heavy metal pollution in plants, which ultimately develops into the systematic pollution of soil–plants. In order to prevent the exacerbation of ecological security by heavy metal pollution, it is essential to exercise caution when discharging industrial waste and to strictly demarcate the study area according to its pollution levels. Furthermore, different treatment measures should be taken according to the degree of pollution.

Meanwhile, the differences between the two models were demonstrated in this study. APCS-MLR relies on the linear assumption of principal components, which may underestimate the nonlinear pollution process, while RF can integrate data from multiple sources, but its “black-box” characteristic limits the direct correlation of chemical characteristics of the pollution sources. Future studies may try the hybrid modeling framework to balance the interpretability and prediction accuracy.

### 3.3. Limitations

The present study exclusively examined representative plants in the designated study area. The plant species are limited; therefore, a subsequent study can include additional species for comparison and analysis of their differences. Furthermore, the model’s accuracy can be enhanced by incorporating additional influencing variables and subdividing the influencing factors of heavy metal pollution in the soil–plant system.

## 4. Materials and Methods

### 4.1. Study Area

The Kalamaili Mountain Nature Reserve, located in the eastern part of Junggar Basin in Xinjiang Uygur Autonomous Region of China, is a very important hoofed reserve in China, with a total area of 14,856.48 km^2^, and its geographic coordinates are 44°36′–46°00′ N, 88°30′–90°00′ E. It has a typical temperate continental arid climate, with hot summers and bitterly cold winters. The administrative area of the reserve covers Fuyun County, Qinghe County, and Fuhai County in the Altay Region and Fukang City, Jimusar County, and Qitai County in Changji Hui Autonomous Prefecture. The plants growing in the reserve mainly include *Anabasis aphylla* L., *Alhagi camelorum* Fisch., *Reaumuria songonica* (PalL) Maxim., *Haloxylon ammodendron* (C. A. Mey.) Bunge., etc., which provide food for a variety of rare wildlife such as *Equus ferus*, *Equus hemionus*, *Gazella subgutturosa*, etc., which live in the protected area.

### 4.2. Sampling and Analysis

#### 4.2.1. Layout of Sample Locations and Design of Sample Collection

**Figure 6:**
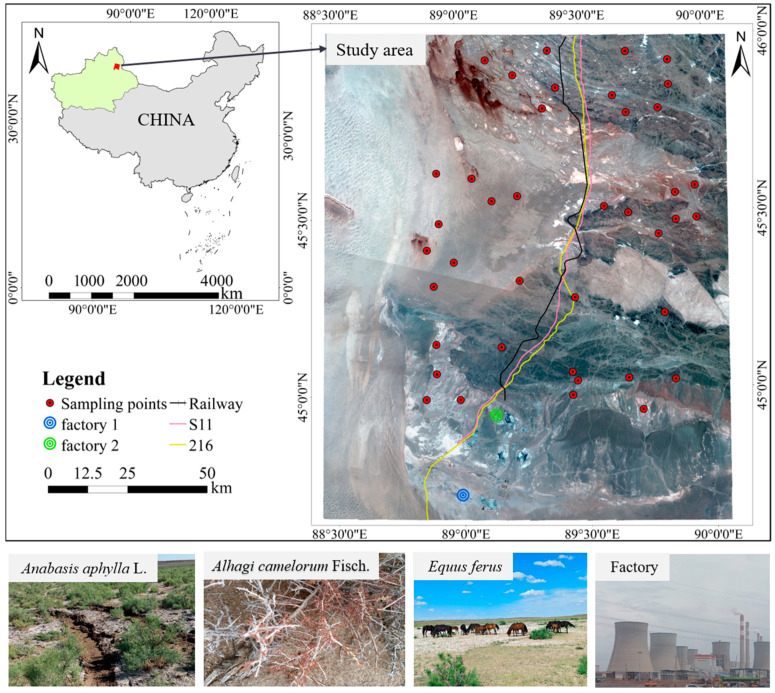
Diagram of the study area. (Map approval number: GS (2023)2767).

In the present study, samples were collected in July 2024 during field surveys in the study area. A total of 36 plants, including 9 *Anabasis aphylla* L., 7 *Alhagi camelorum* Fisch., 6 *Reaumuria songonica* (PalL)Maxim., and 12 *Haloxylon ammodendron* (C. A. Mey.) Bunge., were randomly sampled in 5 m × 5 m sample plots with good coverage and representative of the different plants in the study area, and surface soil samples were collected from 0 to 20 cm at the same locations. The coordinates of each center sample were recorded as a sampling point location using GPS, and the samples were collected (Figure 6), marked and numbered, brought back to the laboratory, dried, pulverized, and finally determined for heavy metal content.

#### 4.2.2. Analysis of Collected Sample

Cd, Pb, Cr, As, Zn, and Cu in the plant samples were determined by inductively coupled plasma mass spectrometry (Agilent 7900) after pretreatment, and Hg in the plant samples was determined by atomic fluorescence spectrometry (Beijing Pudan PF52). The soil samples were pretreated, and then, inductively coupled plasma mass spectrometry (ICP-MS) was used for the determination of Cd, Pb, Cr, Zn, and Cu in the soil samples and atomic fluorescence spectrometry (AFS) was used for the determination of As and Hg in the soil samples. The following sample preparations are required for the determination of heavy metals in plants using both ICP-MS and AFS: weigh about 0.5 g of sample, and add 5 mL of HNO_3_ and 4 mL of H_2_O_2_ microwave digestion and then volume to 50 mL. Before using ICP-MS to determine the heavy metal content of the corresponding element in the soil, it is necessary to weigh about 0.1 g of sample and add 10 mL of mixed acid (HNO_3_: HFO_4_: H_2_O: HF volume ratio of 5:1:4:10) to dissolve the sample. After the perchloric acid fumes are exhausted, add 3 mL of HCL (1:1) to extract it, and then, cool it down to 50 mL, using AFS to determine the heavy metal content of the corresponding elements in the soil before adding 0.5 g of the soil sample to a 25 mL colorimetric tube; adding a little water to wet the sample; adding 5 mL of aqua regia; stoppering it; shaking it well in a boiling water bath for 2 h; then, diluting it with water to scale; shaking it well again; and placing the sample for determination. To ensure the quality of the samples, the repeat sampling rate was kept at 10–15%, and the recovery rate met the analytical quality control requirements.

#### 4.2.3. Data Preprocessing

The data were preprocessed using Excel 2019 (Microsoft, Redmond, WA, USA) software. A principal component analysis and a multifactor regression analysis were performed using SPSS 22.0. (IBM, Armonk, NY, USA). Principal components with eigenvalues of >1 were retained (Kaiser criterion). Arc CIS 10.8 (ESRI, NY Str., Redlands, CA, USA.) was used for the spatial analysis as well as to produce plots, and Python 3.7 was used for modeling and analysis of the random forest model.

### 4.3. Research Methods

In this paper, the potential ecological risk index (RI) and the Nemerow pollution index (NIPI) were used to evaluate the heavy metal pollution of soil samples. In addition, enrichment coefficient and Pearson’s correlation coefficient analyses were used to study the correlation between soil heavy metal content and plant heavy metal content, and APCS-MLR as well as the random forest model were used to analyze the source of plant heavy metal pollution.

#### 4.3.1. Nemerow Pollution Index (*NIPI*)

The NPI takes into account each of the individual assessment factors, while also emphasizing the importance of the most polluted components [88]. The formula is as follows:

(1) $$NIPI=\frac{\sqrt{{\left(P_{ave}\right)}^{2}+{\left(P_{max}\right)}^{2}}}{2}$$

(2) $$P_{i}=\frac{C_{i}}{B_{i}}$$

where *P_ave_* represents the average pollution index of a single element, with the pollution factor used in this study, and *P_max_* is the maximum value of the element. *p_i_* is the pollution index of a single factor, *C_i_* is the measured value of heavy metals, and *B_i_* is the background value of soil in Xinjiang [89]. The specific classification criteria are shown in Table A6.

#### 4.3.2. Potential Ecological Risk Index (*RI*)

The *RI* was proposed by Swedish researcher Lars Hakanson in 1980 and can be used in the assessment of sediments over large areas to synthesize the potential impacts of heavy metals on ecosystems [90]. The index is calculated as follows:

(3) $$RI=\sum_{i=1}^{n}E_{r}^{i}$$

(4) $$E_{r}^{i}=T_{r}^{i}\times P_{i}$$

where *RI* is the integrated potential ecological risk index, *E_r_^i^* is the individual potential ecological risk index of heavy metal *i*, *T_r_^i^* is the toxicity coefficient of heavy metal *i*, and *P_i_* is the single-factor pollution index. According to the literature, the toxicity response coefficients of Cd, Pb, Cr, As, Zn, Cu, and Hg are 2, 5, 1, 30, 5, 40, and 10, respectively [91]. The criteria for categorizing the potential ecological risk index of soil heavy metal pollution are shown in Table A7.

#### 4.3.3. Bioconcentration Factors (BCFs)

*BCF* is the ratio of the elemental content of a part of the plant to the corresponding elemental content of the soil, which to some extent, reflects the ease of elemental transport in the sediment–plant system and indicates the enrichment of heavy metals in the plant [92]. This indicator is calculated as follows:

(5) $$BCF=\frac{C_{p}}{C_{s}}$$

where *C_p_* is the heavy metal content in the aboveground part of the plant and *C_s_* is the heavy metal content in the sediment.

#### 4.3.4. Pearson Correlation Coefficient

The Pearson correlation coefficient was used to assess the correlation between soil heavy metals and plant heavy metals. Pearson’s correlation coefficient is mostly used in factor analysis to calculate the degree of correlation between factors [93]. It is calculated as follows:

(6) $$r=\frac{\sum \left(X_{i}-\bar{X}\right)\left(Y_{i}-\bar{Y}\right)}{\sqrt{\sum {\left(X_{i}-\bar{X}\right)}^{2}\sum {\left(Y_{i}-\bar{Y}\right)}^{2}}}$$

where *X_i_* and *Y_i_* are *i* observations of the two variables, respectively, and $\bar{\;X\;}$ and $\bar{\;Y\;}$ are the mean values of the two variables.

#### 4.3.5. Absolute Principal Component Score–Multiple Linear Regression (APCS-MLR)

The APCS-MLR model was first proposed by Thurston et al. [94]. The principal components of plant indicators were first extracted to provide a basis for the discrimination and quantification of pollution sources. It is calculated as follows:

(7) $${\left(A_{Z}\right)}_{k}=\sum_{j=1}^{P}\left(W_{j}\times Z_{k}\right)$$

(8) $$Z_{k}=\left(C_{k}-\bar{C}\right)/\sigma$$

where (*A_Z_*)*_k_* is the score value of PCA of plant heavy metal elements; *W_j_* is the factor coefficient of *j* PCA, where *j* is the serial number of the principal component obtained in the process of PCA; *Z_k_* is the standardized value of plant heavy metal concentration in the bottom *k* points; *C_k_* is the concentration of plant heavy metal elements in the *k*th points, which is the arithmetic mean of the pollutant concentration; and *σ* is the measured concentration of plant heavy metal elements’ standard deviation (all units: mg·L^−1^).

The (*A_Z_*)*_k_* values must first return the standardized factor scores back to non-standardized absolute principal component scores (APCSs) before they can be used in the analysis of PCs’ contribution to pollution indicators. Their *APCS* is calculated as follows:

(9) $${APCS}_{jk}={\left(A_{Z}\right)}_{jk}-{\left(A_{0}\right)}_{j}$$

(10) $${\left(A_{0}\right)}_{j}=\sum_{i=1}^{q}\left[S_{ij}\times {\left(Z_{0}\right)}_{i}\right]$$

(11) $${\left(Z_{0}\right)}_{i}=\left(0-\bar{C_{i}}\right)/\sigma_{i}$$

where *APCS_jk_* is the *APCS* value of the *j* principal component; (*A_Z_*)*_jk_* is the score value of the *j* PCs; (*A*_0_)*_j_* is the score value of the PCs under the value of 0; *S_ij_* is the factor score coefficient, where *i* is the serial number of the water chemistry factor; (*Z*_0_)*_i_* is the standardized value of the pollutant concentration of the observation point when the pollutant concentration is set to 0; *C* is the arithmetic mean value of the concentration of the *i* phytoalexin; *σ_i_* is the standard deviation of the concentration of the *i* phytoalexin arithmetic mean; and *σ_i_* is the standard deviation of the heavy metal element concentration of the *i* plant. In the formula, *APCS_jk_* is the *APCS* value of the *j* principal component; (*A_Z_*)*_jk_* is the score value of the *j* PCs; (*A*_0_)*_j_* is the score value of the PCs under the value of 0; *S_ij_* is the factor score coefficient, in which *i* is the serial number of the water chemistry factor; (*Z*_0_)*_i_* is the standardized value of the pollutant concentration of the observation point when it is set to 0; *C* is the arithmetic mean value of the concentration of the *i* plant heavy metal; and σ*_i_* is the standard deviation of the heavy metal element concentration of the *i* plant (all units: mg·L^−1^).

#### 4.3.6. Random Forest Model (RF)

The random forest model combines integrated learning with decision tree modeling [95]. Each decision tree in the RF model is constructed by randomly selecting a subset of variables from the original dataset using bootstrap and bagging techniques to avoid overfitting and ensure relatively robust results [96]. However, not all samples were used in the tree generation process. Unused samples omitted from the original dataset are known as out of bag (OOB) samples [97]. The unused out of bag samples will be used to evaluate the performance of the RF model generated by the test.

The data used in the current study include the data-processed heavy metal content of different plants as well as data on five potential sources of contamination, and the infrastructure under consideration includes factory 1, factory 2, railroads, National Highway 216 (henceforth referred to as 216), and the S11 highway (henceforth referred to as S11). The random forest model will build multiple decision models to construct a decision tree by randomly selecting samples from the sample. The above process will be repeated several times to make the final prediction.

## 5. Conclusions

The soils of the Kalamaili Mountain Nature Reserve are seriously polluted by Cd and Hg and have great ecological risks, and the Cr and Hg in the four plants exceed the standard limits and are easily enriched in the plants, causing ecological hazards. The significant differences in Pb, Hg, and As contents in the four species were mainly due to significant differences in *Anabasis aphylla* L., *Reaumuria songonica* (PalL)Maxim., and *Haloxylon ammodendron* (C. A. Mey.) Bunge. Different plants have different enrichment abilities for different heavy metals, and all four plants are easily affected by an enrichment of Cr, Hg, and Cd, among which, *Anabasis aphylla* L. has a stronger response to Cd enrichment. The remaining elements—Cu, Zn, Pb, and As—were not easily enriched in plants. Generally speaking, plants and soil are closely connected, but due to the complex mechanism, the heavy metal content in plants is easily affected by external environmental factors and their own joint influence. The heavy metal pollution of plants in the protected area shows the compound driving characteristic of “industry–traffic”, which affects the ecological environment of the protected area. It is recommended to prioritize the control of industrial emissions and transportation sources and to carry out long-term monitoring to assess the effectiveness of remediation measures, so as to promote improvements in the ecological environment of the protected area.

## Figures and Tables

**Table 1:** Descriptive statistics of heavy metal contents in soil (mg·kg^−1^).

Element	Cr	Cu	Zn	Cd	Pb	Hg	As
Max	95.90	50.30	90.60	0.26	19.3	0.02	21.40
Min	16.30	5.10	30.10	0.03	10.1	0.003	1.730
Median	42.40	21.40	56.60	0.13	17.5	0.009	6.64
Mean	44.38	20.89	55.95	0.13	16.93	0.0093	6.95
CV(%)	35.10	47.85	22.53	45.34	11.03	41.64	59.13
Kurtosis	2.43	0.98	0.45	−0.54	3.35	0.78	5.30
Skewness	1.05	0.70	0.70	0.15	−1.51	0.69	1.93
SD	15.59	10.14	12.65	0.056	1.87	0.00383	4.14
Background in XinJiang [40]	49.30	26.70	68.80	0.12	19.40	0.02	11.20
National Standard [41]	250.00	100.00	300.00	0.600	170.00	3.400	25.00

**Table 2:** Descriptive statistics of heavy metal contents in plants (mg·kg^−1^).

	Element	Cr	Cu	Zn	Cd	Pb	Hg	As
*Anabasis aphylla* L.	Max	480	10.5	18.6	0.31	3.13	0.02	2.65
	Min	16.2	2.56	3.82	0.06	0.44	0.007	0.1
	Median	326	7.01	13	0.15	1.77	0.011	1.56
	Mean	294.24	6.51	11.63	0.17	1.91	0.01	1.53
	CV(%)	60.12	49.59	49.30	54.94	61.23	42.93	71.17
*Alhagi camelorum* Fisch.	Max	135	5.06	6.45	0.1	1.17	0.024	0.93
	Min	11.7	1.98	2.74	0.02	0.18	0.004	0.2
	Median	59.6	3.96	4.72	0.06	0.63	0.01	0.41
	Mean	70.10	3.78	4.89	0.06	0.65	0.01	0.46
	CV(%)	52.44	28.14	24.01	43.96	41.52	56.06	43.62
*Reaumuria songonica* (PalL)Maxim.	Max	175.00	9.08	12.30	0.31	14.13	0.02	1.38
	Min	86.50	3.89	4.90	0.05	0.81	0.01	0.48
	Median	90.00	5.30	8.18	0.08	0.93	0.01	0.83
	Mean	115.46	5.86	8.31	0.12	3.65	0.01	0.92
	CV(%)	34.66	35.85	34.40	87.02	160.72	13.56	39.42
*Haloxylon ammodendron* (C. A. Mey.) Bunge	Max	366.00	6.23	12.10	0.18	0.94	0.01	0.87
	Min	14.70	2.76	2.72	0.04	0.05	0.00	0.06
	Median	34.90	3.55	8.11	0.07	0.36	0.01	0.20
	Mean	63.79	3.85	7.48	0.08	0.39	0.01	0.26
	CV(%)	152.14	25.25	42.77	47.90	56.73	52.01	84.09
Standard		2.00 [42,43]	45.80 [44]	100.00 [44]	0.2 [45]	3.00 [46]	0.01 [47,48]	5.00 [49]

**Table 3:** Characterization of NIPI.

	Cr	Cu	Zn	Cd	Pb	Hg	As
NIPI	1.516	1.445	1.095	1.696	0.935	0.916	1.421
level	Light Pollution	Light Pollution	Light Pollution	Light Pollution	Alert	Alert	Light Pollution

**Table 4:** Characterization of a comprehensive ecological risk index for soil heavy metals.

	Cr	Cu	Zn	Cd	Pb	Hg	As
RI	70.28	154.81	31.84	1205.00	170.00	844.71	243.62
Level	Low Risk	Moderate Risk	Low Risk	Very High Risk	Moderate Risk	Very High Risk	Low Risk

**Table 5:** Principal component analysis of APCS-MLR.

	PC1	PC2
Cr	0.76	0.44
Cu	0.64	0.64
Zn	0.79	0.26
Cd	0.78	−0.23
Pb	0.01	0.74
Hg	0.13	0.77
As	0.66	0.70

Note: PC1 refers to the first principal component, which explains the direction of the largest variance in the data, usually representing the most dominant pollution source or influencing factor; PC2 refers to the second principal component, which explains the second largest direction in the remaining variance, usually representing the secondary pollution source or influencing factor.

**Table 6:** Evaluation of the effectiveness of RF model prediction.

Element	Training Test			Testing Set			RPD
	R^2^	RMSE	MAE	R^2^	RMSE	MAE	
Cr	0.76	66.81	41.57	0.69	58.88	42.91	1.78
Cu	0.81	0.94	0.69	0.82	0.45	0.42	2.35
Zn	0.72	2.08	1.81	0.54	1.94	1.67	1.48
Cd	0.81	0.03	0.02	0.86	0.02	0.02	2.66
Pb	0.85	1.07	0.46	0.75	0.19	0.16	2.01
Hg	0.81	0.002	0.002	0.71	0.002	0.002	1.86
As	0.81	0.31	0.23	0.81	0.16	0.14	2.27

## Data Availability

The data presented in this study are available from the corresponding author on request. The data are not publicly available due to the ongoing nature of the research.

## Abbreviations

The following abbreviations are used in this manuscript:

Cr	Chromium
Cu	Copper
Zn	Zinc
Cd	Cadmium
Pb	Lead
Hg	Mercury
As	Arsenic
Max	Maximum value
Min	Minimum value
Median	Median value
Mean	Mean value
CV	Coefficient of variation
SD	Standard deviation
NIPI	Nemerow pollution index
Pi	Individual pollution index
RI	Comprehensive ecological risk index
PC1	Principal component 1
PC2	Principal component 2
Factor 1	The first impact factor
Factor 2	The second impact factor
Factor 3	The third impact factor
R^2^	Coefficient of determination
RMSE	Root mean square error
MAE	Mean absolute error
RPD	Relative percent difference
S11	S11 highway
216	National Highway 216

## Appendix A

### Appendix A.1

**Table A1:** Significance of soil heavy metal content in comparison with background values in Xinjiang.

	Cr	Cu	Zn	Cd	Pb	Hg	As
*p*-value	0.9183	0.9934	1	0.1994	1	1	1

**Table A2:** Significance of plants’ heavy metal content in comparison with the standard.

	Cr	Cu	Zn	Cd	Pb	Hg	As
*p*-value	0.0001	0.0001	0.00001	0.00001	0.00001	0.3699	0.00001

**Table A3:** Significance test for heavy metal content among plants.

	Cr	Cu	Zn	Cd	Pb	Hg	As
*p*-value	0.1210	0.0699	0.3220	0.0693	0.001	0.0054	0.0019

**Table A4:** Significance test for heavy metal (Pb, Hg, and As) content among plants.

		*Alhagi camelorum* Fisch.	*Anabasis aphylla* L.	*Reaumuria songonica* (PalL)Maxim.	*Haloxylon ammodendron* (C. A. Mey.) Bunge
Pb	*Alhagi camelorum* Fisch.	1	0.5119	0.4507	0.5330
	*Anabasis aphylla* L.	0.5119	1	1	0.0079
	*Reaumuria songonica* (PalL)Maxim.	0.4507	1	1	0.0063
	*Haloxylon ammodendron* (C. A. Mey.) Bunge	0.5330	0.0079	0.0063	1
Hg	*Alhagi camelorum* Fisch.	1	1	0.6816	0.4297
	*Anabasis aphylla* L.	1	1	1	0.0777
	*Reaumuria songonica* (PalL)Maxim.	0.6816	1	1	0.0098
	*Haloxylon ammodendron* (C. A. Mey.) Bunge	0.4297	0.0777	0.0098	1
As	*Alhagi camelorum* Fisch.	1	1	0.9283	0.2660
	*Anabasis aphylla* L.	1	1	1	0.0145
	*Reaumuria songonica* (PalL)Maxim.	0.9283	1	1	0.0096
	*Haloxylon ammodendron* (C. A. Mey.) Bunge	0.2660	0.0145	0.0096	1

**Table A5:** Coefficients of the linear regression model.

Element		Non-Standardized Coefficient		Standardized Coefficient	t	Significance	R^2^
		B	Standard Error	Beta			
Cr	Constant	−85.543	21.803		−3.923	0.001	0.776
	S1	93.004	10.655	0.755	8.729	0.000	
	S2	58.001	10.655	0.471	5.444	0.000	
Cu	Constant	1.487	0.323		4.603	0.000	0.810
	S1	1.208	0.158	0.609	7.648	0.000	
	S2	1.33	0.158	0.671	8.426	0.000	
Zn	Constant	1.934	0.815		2.374	0.025	0.674
	S1	2.991	0.398	0.783	7.51	0.000	
	S2	1.091	0.398	0.286	2.741	0.011	
Cd	Constant	0.026	0.015		1.664	0.107	0.642
	S1	0.055	0.008	0.791	7.241	0.000	
	S2	−0.014	0.008	−0.2	−1.829	0.078	
Pb	Constant	−0.387	0.642		−0.602	0.552	0.527
	S1	−0.082	0.314	−0.033	−0.261	0.796	
	S2	1.864	0.314	0.746	5.941	0.000	
Hg	Constant	0.005	0.001		3.914	0.001	0.607
	S1	0.001	0.001	0.156	1.364	0.184	
	S2	0.004	0.001	0.781	6.823	0.000	
As	Constant	−0.43	0.07		−6.147	0.000	0.916
	S1	0.405	0.034	0.628	11.838	0.000	
	S2	0.468	0.034	0.726	13.676	0.000	

Note: The B value is the regression coefficient and intercept (constant term); S1 and S2 denote the regression coefficients of source 1 and source 2 on the factors of each heavy metal element, respectively; t denotes the value of the *t*-test process, which is the result of the division of B with the standard error; and R2 denotes the decidability factor.

**Table A6:** The NIPI classification criteria.

Level	Value
Clean (Grade I)	NPI < 0.7
Alert (Grade II)	0.7 ≤ NIPI < 1
Light Pollution (Grade III)	1 ≤ NIPI < 2
Moderate Pollution (Grade IV)	2 ≤ NIPI < 3
Severe Pollution (Grade V)	3 ≤ NIPI

**Table A7:** Criteria for evaluating potential ecological risk indices.

$E_{r}^{i}$	RI	Level
$E_{r}^{i}$ < 40	RI < 150	Low Risk
40 ≤ $E_{r}^{i}$ < 80	150 ≤ RI < 300	Moderate Risk
80 ≤ $E_{r}^{i}$ < 160	300 ≤ RI < 600	High Risk
160 ≤ $E_{r}^{i}$ < 320	600 ≤ RI < 1200	Very High Risk
$E_{r}^{i}$ ≥ 320		Extremely High Risk
